# Gamma Radiation Induced In-Vitro Mutagenesis and Isolation of Mutants for Early Flowering and Phytomorphological Variations in Dendrobium ‘Emma White’

**DOI:** 10.3390/plants11223168

**Published:** 2022-11-18

**Authors:** Rubina Sherpa, Ramgopal Devadas, Sadashiv Narayan Bolbhat, Tukaram Dayaram Nikam, Suprasanna Penna

**Affiliations:** 1 Department of Botany, Annasaheb Awate College, Manchar 410503, India; 2 Indian Council of Agricultural Research-National Research Centre on Orchids, Pakyong 737106, India; 3 Department of Botany, Savitribai Phule Pune University, Pune 411007, India; 4 Nuclear Agriculture and Biotechnology Division, Bhabha Atomic Research Centre, Mumbai 400085, India; 5 Amity Institute of Biotechnology, Amity University Mumbai, Mumbai 410206, India

**Keywords:** orchids, mutation, genetic markers, polymorphism, random amplified polymorphic DNA

## Abstract

In vitro mutagenesis offers a feasible approach for developing new orchid cultivars through genetic manipulation. In the present study, protocorm-like bodies (PLBs) were exposed to gamma rays (10, 20, 40, 60, 80 Gy) to study in vitro growth responses and induction of mutants in Dendrobium ‘Emma White’. Both proliferation and regeneration of PLBs decreased progressively with increasing doses, except for a significantly enhanced growth response at 10 Gy. The optimal dose of gamma radiation for mutagenesis was found in the range 10 to 25 Gy based on the growth reduction curve. Analysis using a high-throughput cell analyzer revealed a significant reduction in nuclear DNA content at > 40 Gy doses. At 10 Gy treatment, the growth attributes, such as root length, plant height and leaf number, were significantly increased by 36%, 26% and 20%, respectively, compared to the control. This increase was significant over other tested doses as well. Testing of random amplified polymorphic DNA markers revealed the presence of detectable polymorphism among gamma mutant plantlets with a polymorphism information content value at 0.41. The gamma-ray-induced earliness in flower development was observed within 294 days post ex vitro growth of 10 Gy mutant compared to the control plants flowered after 959 days. Our results highlight the significance of gamma radiation in inducing enhanced growth, morphological variations and early floral initiation in Dendrobium, providing a basic framework for mutation breeding and improvement of orchids.

## 1. Introduction

Dendrobium is the second largest genus after Bulbophyllum in the Orchidaceae family. Dendrobiums were used as rich medicinal plants in many old-world countries, including both China and India [1]. Dendrobiums also have potential demand all over southeast Asia and other tropical parts for exports due to the wide range of choices for flower color, shape, texture and longevity [2]. Worldwide Dendrobium marketing and trade occur broadly as cut flowers and potted plants. Dendrobium occupied among the top ten orchid taxa of commercially traded and propagated live plants at 2.3% (hybrids) and 3.4% (species) between 2006 and 2015 [3]. Thailand is the largest producer and exporter of Dendrobium, with 70% of the total 2.1 billion cut flowers exported globally [4]. However, only a few Dendrobium varieties dominate export trade from southeast Asian countries, and the majority of these varieties are genetically derived from *Dendrobium phalaenopsis* species [5]. ‘Sonia’ cultivar from Thailand and ‘Uniwai’ cultivar from Hawaii occupy 70% of total Dendrobium production [6,7], indicating the limited choice in varieties. Developing new genetic stocks with desirable traits will be helpful in meeting the demand for new Dendrobium varieties across international and domestic markets [8,9].

Development of a large number of new Dendrobium varieties is constrained by compatibility barriers within inter-sectional crossing in the genus and lack of advanced segregating progenies to construct genetic and linkage maps [8,10]. Further, linkage drag and negative linkage for promising characters, apart from prolonged juvenile phase [11] and mortality at ex vitro hardening [12], hinder the selection process. The absence of natural hybrids from the center of origin (Australia) also supports the crossability problems in Dendrobium [13], and even self-pollination leads to drastic adverse effects on several traits that were unresponsive to selection in this species [14]. Conventional breeding programs involving traditional hybridization to transfer desirable traits are laborious and take 12–13 years to develop new cultivars [15]. Hence, application of mutation breeding can offer quick and better solutions to overcome such inherent pre- and post-zygotic barriers to develop new vegetatively propagated Dendrobium cultivars. A wide range of trait-specific mutant varieties from plant structure to biotic and abiotic stress resistance with high yield have been successfully achieved through mutation breeding in other crops [16,17].

Among orchids, the initial studies on mutagenesis were conducted in Cattleya using gamma rays [18]. The changes in flower color pigmentation and flower size were reported in Dendrobium ‘Sonia’ cultivar through gamma radiation [19]. Induced mutations in orchid breeding for genetic improvement are restricted to a few genera; however, from a breeding point of view for developing new cultivars, it is essential to determine the correct mutagen, the optimum mutagen dose and the choice of developmental stage for treatment. Such studies will provide a baseline for other mutation breeding work for orchid varietal development. In the present study, we have studied the effect of gamma-radiation-induced in vitro and ex vitro growth responses and mutagenic changes at the cellular and genetic level in Dendrobium hybrid ‘Emma White’.

## 2. Results

### 2.1. Growth Response of Protocorm Like Bodies (PLBs) to Different Doses of Gamma Radiation

**Figure 1:**
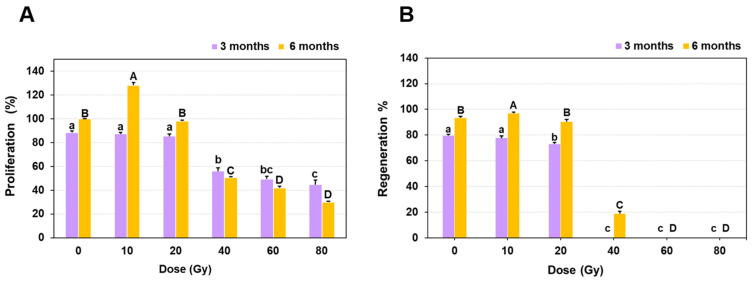
Growth response of protocorm-like bodies (PLBs) of Dendrobium after gamma irradiation. PLBs of Dendrobium ‘Emma White’ were irradiated with gamma ray doses of 10, 20, 40, 60 and 80 Gy and incubated at 22 ± 2 °C and 65–70% relative humidity with 16 h photoperiod. An unirradiated set of PLBs was maintained as control. After three and six months of irradiation, growth response of irradiated PLBs based on proliferation percentage (**A**) and regeneration percentage (**B**) was recorded for each treatment. Data represent mean values ± standard error (*n* = 10). Means with different lower-case letters (a, b, c, d) are significantly different at *p* < 0.05 (Duncan’s Multiple Range Test).

The fresh weight of protocorm-like bodies (PLBs) of Dendrobium ‘Emma White’ measured a month after irradiation (MAI) ranged from 448.2 mg (10 Gy) to 209.1 mg (80 Gy), indicating a gradual decrease in tissue biomass with increasing doses of gamma irradiation as compared to the control (Table 1). However, the reduction by ~ 60 to 70% in tissue fresh weight was more evident at doses > 40 Gy. At 3 MAI, no significant differences were observed for proliferation among PLBs irradiated at 10 and 20 Gy doses (Figure 1A). In contrast, the proliferation was reduced drastically by 35 to 50% for PLBs treated with gamma ray doses of 40, 60 and 80 Gy compared to the control. At 6 MAI, a 28% increase in proliferation was observed at 10 Gy, in contrast to doses > 40 Gy, where the proliferation reduced significantly by 70 to 90% compared to the control (Figure 1A). A similar effect on the regeneration capacity of PLBs was observed at subsequent stages of growth and development. Initially, there was no evidence of PLB regeneration into shoots at higher doses, except for 10 and 20 Gy, where the regeneration percentage was comparable to the control (Figure 1B). At 6 MAI, PLBs exposed to 10 Gy radiation showed an increase (4%) in regeneration as compared with the control. However, a lower regeneration response at a gamma dose of 40 Gy (18.86%) with suppressed growth was observed at 60 and 80 Gy doses, indicating that PLBs are sensitive to higher doses of gamma radiation (Figure 1B). The average number of days required for initiation of leaf primordium from PLBs irradiated at 10 and 20 Gy was 36.2 days and 38.8 days compared to the control (32.2 days). On the other hand, a prolonged delay in the initiation of leaf primordium at a dose of 40 Gy (106.4 days) was observed, indicating an adverse effect of gamma irradiation on PLBs differentiation and the developmental process.

**Figure 2:**
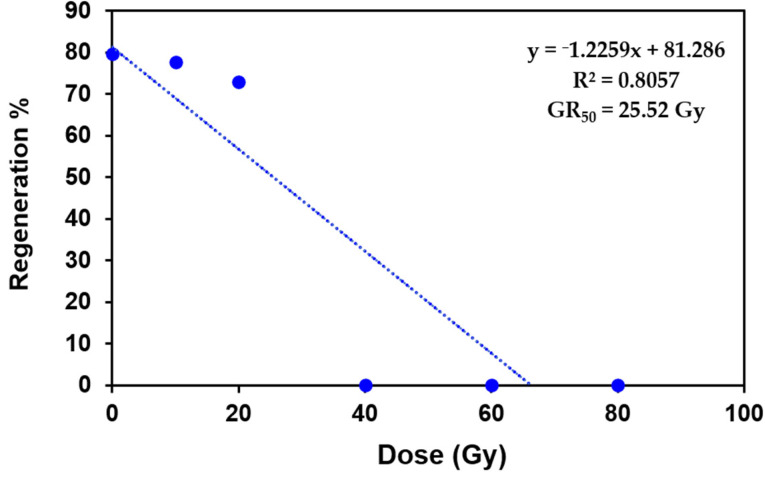
Linear regression analysis to estimate growth reduction dose (GR_50_) of gamma-irradiated protocorm-like bodies of Dendrobium ‘Emma White’. Shoot regeneration response of PLBs irradiated with gamma rays of 10, 20, 40, 60 and 80 Gy after three months of radiation treatment was used to estimate GR_50_. The mean values of regeneration percentage were analyzed using linear regression equation (y = mx + c).

In the present study, all the PLBs of Dendrobium ‘Emma white’ irradiated with gamma ray doses of 10, 20, 40, 60 and 80 Gy survived with no browning or desiccation, making it difficult to estimate the lethal dose (LD_50_). Hence, the radiation sensitivity of PLBs towards gamma rays was assessed based on the growth reduction dose (GR_50_), at which the growth of PLBs reduced by 50% [20]. The GR_50_ was estimated to be 25.52 Gy based on the initial regeneration response of irradiated PLBs (Figure 2), suggesting an optimal dose range of 10 to 25 Gy for irradiation of Dendrobium ‘Emma White’ PLBs.

### 2.2. Nuclear DNA Content Estimation and Cell Cycle Analysis in Irradiated PLBs by High-Throughput Cell Analyser (HTCA)

**Figure 3:**
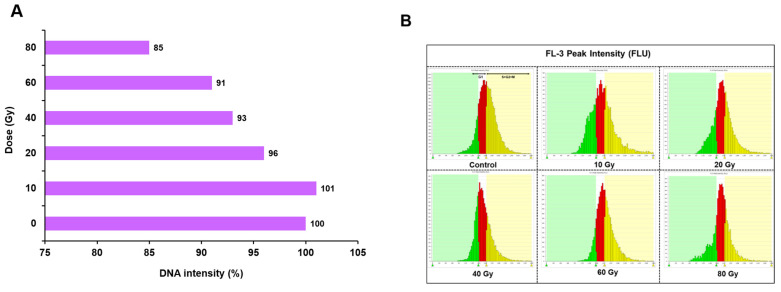
High-throughput-cell-analyser (HTCA)-based nuclear DNA content estimation and cell cycle analysis in protocorm-like bodies (PLBs) of Dendrobium under different gamma radiation treatments. PLBs of Dendrobium ‘Emma White’ were irradiated with gamma ray doses of 10, 20, 40, 60 and 80 Gy and incubated at 22 ± 2 °C and 65–70% relative humidity with 16 h photoperiod. After five months of treatment, nuclei were isolated from irradiated and control PLBs to estimate the nuclear DNA content based on the average fluorescence intensity of nuclei generated by HTCA, (**A**) Percentage of DNA intensity calculated based on fluorescence intensity of nuclei under different radiation treatments of 10, 20, 40, 60 and 80 Gy, including control using HTCA, (**B**) FL-3 fluorescence intensity histogram of nuclei isolated from PLBs, incubated with propidium iodide overnight at 4 °C in dark conditions. Gate between 0–1200 indicates dead cells (green), 1201–1600 live non-dividing cells in G1 phase (red) and 1601–2800 dividing cells in S, G2 and M phase (yellow) of cell cycle. The 488 nm laser-excited propidium iodide dye (FL-3) intercalates DNA and facilitates enumeration of DNA per nuclei number.

The estimation of nuclear DNA content based on the average fluorescence intensity ranged from 101 to 85%, with a maximum of 101%, followed by 96% under 10 and 20 Gy doses, respectively (Figure 3A). In contrast, gamma-irradiated PLBs at higher doses showed a significant decrease in DNA content, with a maximum (15%) reduction at 80 Gy. Similarly, at 40 and 60 Gy, the DNA content of PLBs was reduced by ~7 to 9% compared to the control. The results indicated that gamma radiation exerted more effect in PLBs irradiated at higher doses, as was evident from the average fluorescence intensity of nuclear DNA generated by high-throughput cell analyzer (HTCA) (Supplementary Table S1). A histogram of the FL-3 fluorescence intensity peak distribution of nuclei isolated from gamma-irradiated PLBs after five months of irradiation is shown in Figure 3B. The percentage of cell count at the S + G2M phase in gamma-irradiated PLBs was decreased by ~ 14 to 50% with increasing irradiation doses of 20, 40, 60, and 80 Gy, indicating inhibition of cell cycle progression at the higher dose. Unlike other treatments, the cell count percentage in the 10 Gy treatment was increased by 2% at the S + G2M phase compared to the control treatment used as the benchmark value (Supplementary Table S1).

### 2.3. Frequency and Spectrum of Variation among Gamma-Irradiated Population of Dendrobium ‘Emma White’

**Figure 4:**
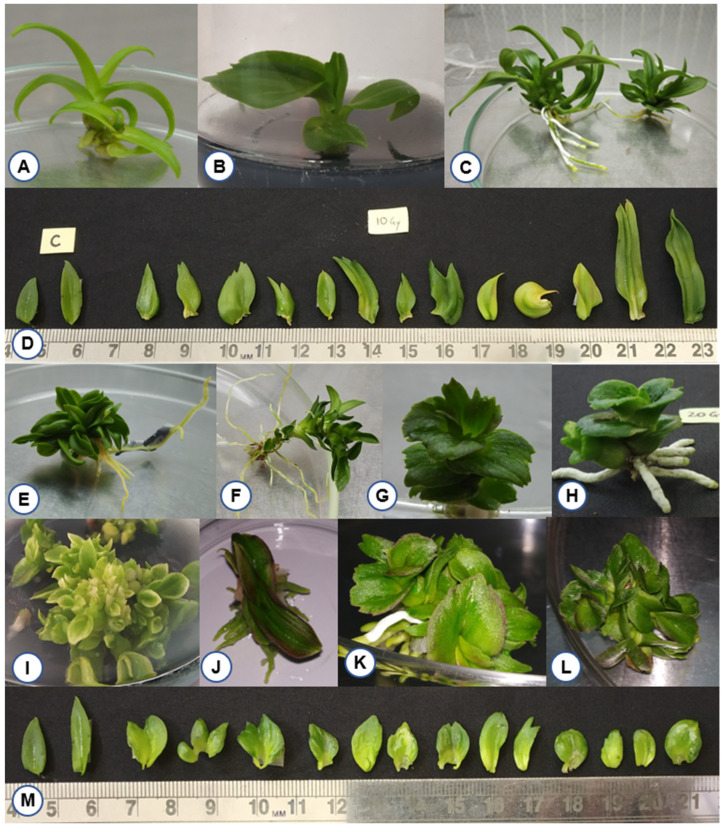
Morphological variations among in vitro plantlets regenerated from gamma-irradiated protocorm-like bodies (PLBs) of Dendrobium ‘Emma White’. PLBs of Dendrobium ‘Emma White’ were irradiated with gamma ray doses of 10, 20, 40, 60 and 80 Gy and incubated at 22 ± 2 °C and 65–70% relative humidity with 16 h photoperiod. Seven to eight months post irradiation, alterations in growth and structure of shooting pattern were observed at 10, 20 and 40 Gy doses. (**A**) Control unirradiated plantlet, (**B**–**D**) leaf variations at 10 Gy, (**E**–**H**) leaf variations at 20 Gy, (**I**–**M**) Leaf variations at 40 Gy. Morphological variation observed in leaf distribution pattern (**B**,**C**,**E**); leaf margin, midrib, apex, shape, and color (**D**,**M**); leaf margin (**G**,**J**,**K**,**L**); leaf color (**I**); leaf texture (**H**); multiple shoots formation (**F**).

During the initial period of in vitro regeneration, plantlets developed from gamma-irradiated PLBs did not exhibit any morphological variation. However, after seven to eight months of irradiation, variation in the leaf shape and shooting pattern was observed. Alterations in leaf morphology (yellowing, asymmetrical and oval to cordate shapes) was observed at 10, 20 and 40 Gy doses (Figure 4). However, no shoot regeneration was observed from gamma-irradiated PLBs at 60 and 80 Gy. The highest frequency of variation (51.6%) was observed in the gamma-irradiated population of 40 Gy (Table 2), followed by the lower frequencies in PLBs irradiated at 20 Gy (9.5%) and 10 Gy (7%). The maximum spectrum of variation (10) was observed at 40 Gy compared to the spectrum of variation of 7 at lower doses (10, 20 Gy). The number of plantlets regenerated was reduced by 80% at 40 Gy compared to the highest number of regenerated plantlets at 10 Gy and the control. Among the mutagenic changes observed for morphological traits in the gamma-irradiated population, the variations induced in leaf margin (serration) were higher in proportion than other leaf variations (leaf margin > leaf apex > leaf vein > leaf shape) (Table 2).

### 2.4. Phenotypic Variation among In Vitro Plantlets Derived from Gamma-Irradiated PLBs

**Figure 5:**
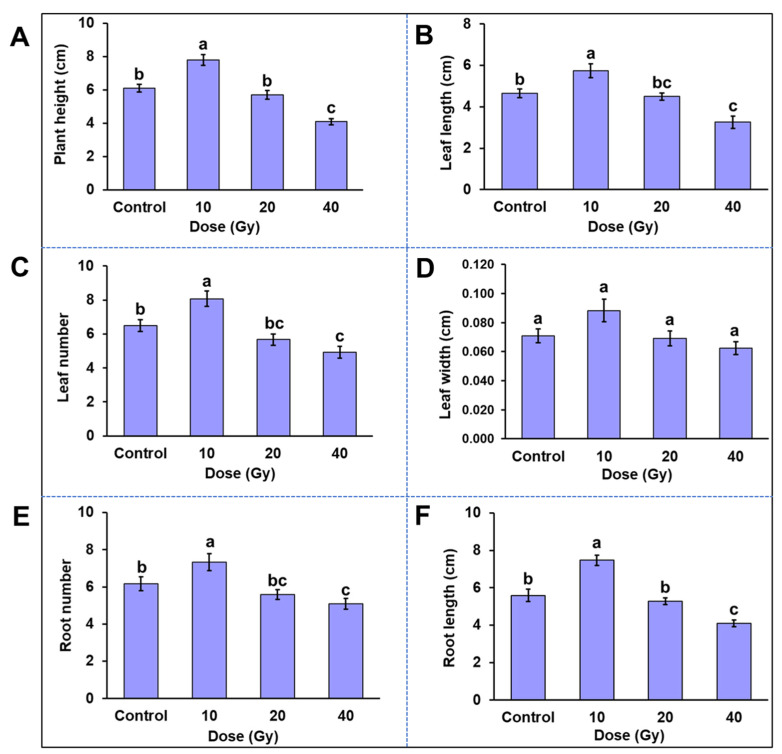
Variations in phenotypic characters of in vitro plantlets at 10, 20 and 40 Gy gamma radiation treatments. Protocorm-like bodies (PLBs) of Dendrobium ‘Emma White’ were irradiated with gamma rays of 10, 20, 40, 60 and 80 Gy and incubated at 22 ± 2 °C and 65–70% relative humidity with 16 h photoperiod. After 11–12 months of irradiation, differential phenotype of regenerated plantlets at 10, 20 and 40 Gy doses were recorded concerning plant growth attributes (**A**) plant height, (**B**) leaf length, (**C**) leaf number, (**D**) leaf width, (**E**) root number, (**F**) root length and root width. No significant difference in root width was observed under all the responsive treatments. Data represent mean value ± standard deviation (*n* = 10). Mean values with different lower-case letters (a, b, c, d) are significantly different at *p* < 0.05 (Duncan’s Multiple Range Test).

A significant effect of gamma radiation was observed on plant growth attributes viz., plant height, leaf length, leaf number, leaf width, root number and root length (Figure 5). Except for the 10 Gy treatment, all the other gamma irradiation doses showed a dose-dependent decrease in plant growth. The lowest radiation dose induced a significant difference in growth of plantlets when compared to the control. Compared with the control, the root length was increased by 36%, plant height by 26% and leaf number by 20% in the 10 Gy treatment. However, no significant differences were observed for root width in all treatments. The overall decrease in plant growth attributes at dose 20 Gy ranged from a minimum reduction in leaf width by 2% to a maximum reduction in leaf number by 9% compared to the control. At a higher dose of 40 Gy, significant differences were observed in plant height, leaf number and length, root number and length. The plant height was reduced by 33%, followed by a 30% reduction in leaf length at a 40 Gy dose compared to the control.

### 2.5. Genetic Analysis of Regenerated Mutants Based on Random Amplified Polymorphic DNA Markers

**Figure 6:**
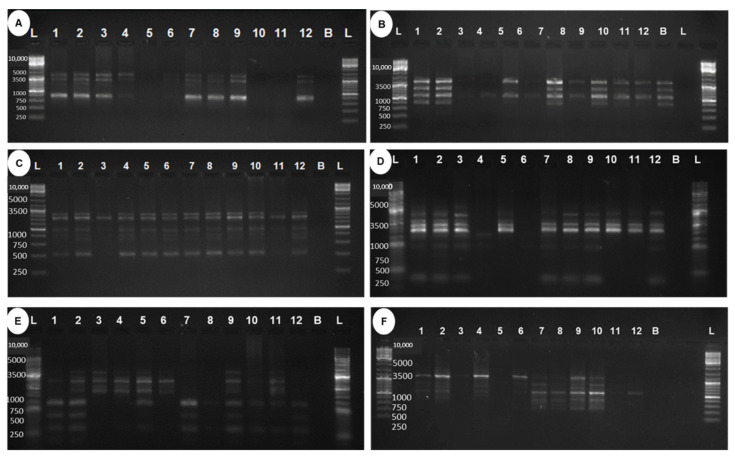
Random amplified polymorphic DNA (RAPD) polymorphism in gamma-ray-induced mutants of Dendrobium ‘Emma White’. Total genomic DNA was isolated from in vitro young leaves of control and mutant plantlets using a modified CTAB method for RAPD marker level study to analyze the radiation-induced genetic variability in ‘Emma White’. Twenty-eight decamer RAPD primers were used, out of which six primers (OPB 12, OPA 4, OPAW 13, OPAW 17, OPB 5, OPF 1) that produced clear and scorable bands were used to generate RAPD binary matrix. Bands were scored as the presence (1) or the absence (0) for control and mutant DNA samples. DNA ladder (lane L); control 1 (lane 1); control 2 (lane 2); 10 Gy_1 (lane 3); 10 Gy_2 (lane 4); 10 Gy_3 (lane 5); 10 Gy_4 (lane 6); 20 Gy_1 (lane 7); 20 Gy_2 (lane 8); 20 Gy_3 (lane 9); 40 Gy_1 (lane 10); 40 Gy_2 (lane 11); 40 Gy_3 (lane 12); blank without DNA to check cross-contamination (lane B). Primers: (**A**) OPB12; (**B**) OPA4; (**C**) OPAW13; (**D**) OPAW17; (**E**) OPB5; (**F**) OPF1. GeneRuler1 Kb DNA Ladder.

**Figure 7:**
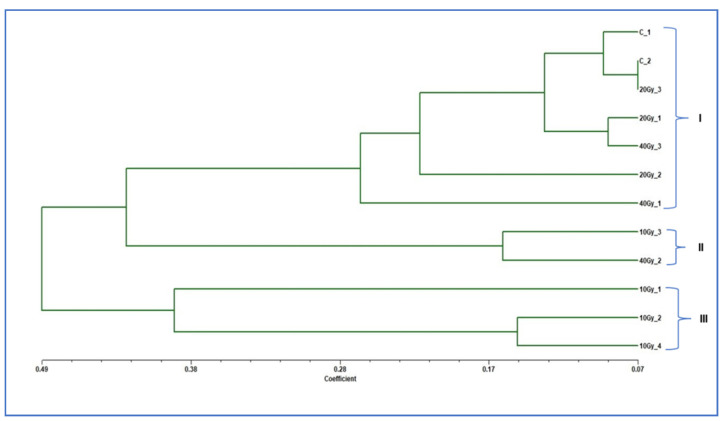
Dendrogram based on random amplified polymorphic DNA (RAPD) binary data. Dendrogram constructed using Nei’s dissimilarity coefficient by UPGMA-based clustering method showed three major clusters (I, II, III), with a cophenetic correlation coefficient of 0.89. Cluster I included mutants of 20 Gy_1, 2, 3 and 40 Gy_1, 3, including two controls of ‘Emma White’ C_1, 2; cluster II included mutants of 10 Gy_3 and 40 Gy_2, whereas cluster III included three mutants of 10 Gy_1, 2, 4.

The genetic divergence among gamma-irradiated mutants of ‘Emma White’ with respect to control plants was analyzed based on random amplified polymorphic DNA (RAPD) markers (Figure 6). A total of 33 bands were generated using six RAPD primers, and the number of bands ranged from four to eight, with an average of 5.50 bands per primer, of which four bands were monomorphic and the remaining twenty-nine bands were polymorphic (Table 3). The percentage of polymorphism was found to be 100% for all the primers except for OPAW13, 33.33%, and an average polymorphism of 88.89%. The polymorphism information content (PIC) value of different RAPD primers ranged from 0.15 to 0.5, with an average of 0.41 (Table 3). Among all six RAPD primers, two primers (OPB5 and OPF1) were found with a PIC value at 0.5, indicating a highly informative nature for the study of gamma-ray-induced mutants. OPAW13 showed the minimum PIC value at 0.15. NEI72 dissimilarity-coefficient-based genetic distance ranged from 0.07 to 0.89, with an average distance of 0.37, indicating a greater range of genetic diversity among gamma-irradiated mutants of ‘Emma White’ (Table 4). A maximum genetic distance of 0.89 was observed between mutants generated at 40 Gy_2 and 10 Gy_2. In contrast, the lowest distance of 0.07 was obtained between 20 Gy_3 and Control_2, followed by the distance of 0.09 between two control plants. The average genetic distance between the mutants and the control was found to be in the order of 0.16 (20 Gy) < 0.26 (40 Gy) and 0.37 (10 Gy), indicating the most distant genetic divergence among the 10 Gy mutants with respect to the control. A dendrogram constructed by the UPGMA-based clustering method showed three major clusters (I, II, III) with a genetic dissimilarity coefficient of 0.40 and with a cophenetic correlation coefficient of 0.82 [21]. Cluster I included mutants of 20 Gy and 40 Gy, including controls of ‘Emma White’; cluster II included mutants of 10 Gy and 40 Gy, whereas cluster III included three mutants of 10 Gy (Figure 7).

### 2.6. Leaf Variation among 10 Gy Mutant Population

**Figure 8:**
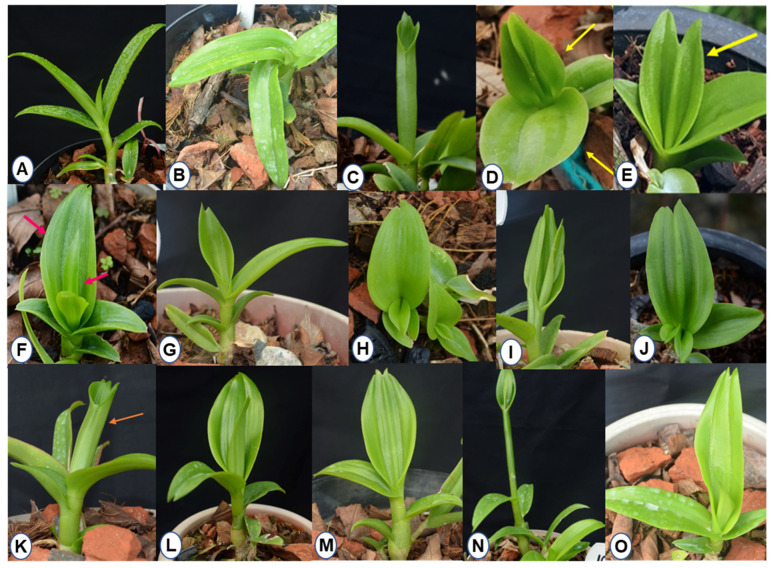
Morphological variations in 10 Gy mutant plants of Dendrobium ‘Emma White’ after 7–8 months of ex vitro transfer of well grown in vitro plantlets. (**A**) Control ((**B**):10/29) Leaves twisted in an anti-clockwise direction from the base. ((**C**):10/5) Closed leaf with marginal serrations around apex. ((**D**):10/85) Deformed leaf emerged from base of another fused leaf. ((**E**):10/35) Bilobed leaf with a deep notch, leaves fused to form two separate midribs. ((**F**):10/17) Asymmetric ovate leaf with two midribs, two middle leaves emerged from fourth leaf. ((**G**):10/4) Broad leaf with two midribs, notch at apex, folded base. ((**H**):10/37) Deltate (triangular leaf) with uneven leaf growth. ((**I**):10/33) Third and fourth leaf fused with other leaves. ((**J**):10/35) Two leaves emerged from the base of another bilobed leaf. ((**K**):10/111) Closed leaf with marginal tooth or serrations around apex. ((**L**):10/21) Oval-shaped leaf with two midribs. ((**M**):10/79) Two midribs with slight elevation, small pseudo-bulb like appearance at the base. ((**N**):10/41) Long needle-shaped leaf (12.4 × 1.3 cm) opened at top. ((**O**):10/16) Leaf with two midribs, twisted, three pointed apexes.

After 7–8 months of ex vitro growth and development, morphological variations were observed among plants regenerated from 10 Gy-irradiated PLBs compared to the control (Figure 8). The differences were more apparent in leaf structure and arrangement of individual shoots. These variations ranged from double apexed leaves, fused leaves, multiple and raised midribs, serrated leaf tips, bilobed leaves, twisted leaves and broad/elliptic/ovate/triangular/linear-shaped leaves. Under a 10 Gy irradiation dose, 13 leaf mutants (10/4, 10/5, 10/16, 10/17, 10/21, 10/29, 10/33, 10/35, 10/37, 10/41, 10/79, 10/85, 10/111) were observed with morphological traits distinct from the control (Figure 8).

### 2.7. Isolation of Early Flowering Mutant among 10 Gy Mutant Population

**Figure 9:**
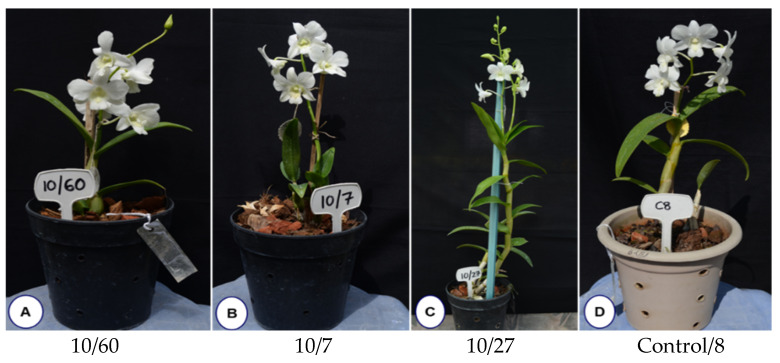
Gamma-radiation-induced early flowering mutant lines recovered from gamma-irradiated protocorm-like bodies of Dendrobium ‘Emma White’ at dose 10 Gy (**A**–**C**) and control (**D**).

**Figure 10:**
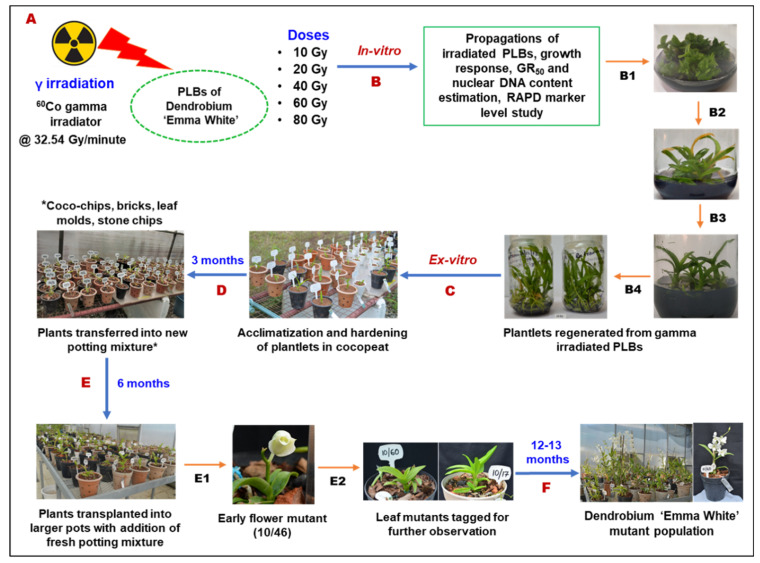
Workflow of gamma irradiation-induced mutagenesis in Dendrobium ‘Emma White’. (**A**) Protocorm-like bodies (PLBs) generated from the shoot-tip of ‘Emma White’ was irradiated with different doses of gamma rays (10, 20, 40, 60, 80 Gy); (**B**) Irradiated PLBs were transferred onto the fresh culture medium and incubated in a culture room at 22 ± 2 °C and 65–70% relative humidity with 16 h of photoperiod. After three and six months of gamma irradiation, growth response of PLBs was recorded based on proliferation and regeneration percentage; nuclei isolated from PLBs irradiated with gamma rays were used for estimation of nuclear DNA content based on fluorescence intensity using high throughput cell analyser (HTCA); genetic diversity of in-vitro plantlets regenerated from gamma irradiated PLBs at dose 10, 20, and 40 Gy were analysed using random amplified polymorphic DNA (RAPD) markers; (**C**) Ex-vitro acclimatization and hardening of well rooted mutant and control plantlets in coco-peat; (**D**) After three months, plants were re-potted into a mixture of media containing coco-chips, brick pieces, leaf molds, and stone chips in 1:2:2:1 ratio; (**E**) After six months, plants were transplanted into larger pots with fresh potting mixture; (**E1**) First flowering in mutant 10/46 was observed within 294 days of ex-vitro growth; (**E2**) Morphological variations were observed in leaves of 10 Gy mutant plants as compared to control; (**F**) After twelve to thirteen months, well-grown Dendrobium ‘Emma White’ mutant population were established with early flowering at dose 10 Gy.

The first early flowering mutant (10/46) was isolated within 294 days of ex vitro transfer compared to several years required for flower development in control plants. This mutant plant was observed with a plant height of 7.2 cm, with the growth appearing stunted and with no change in flower color. In addition, alteration in the structure of the flower was observed as dorsal and lateral sepals fused with no petals. The lip of the early mutant flower appeared slightly rounded with wavy edges and longer side lobes compared to the flower of the mother plant (Supplementary Figure S1). The flower bud formation was initiated at 224 days of ex vitro growth, with a potted vase life of 50 days, as indicated by the number of days to withering. Among the gamma-ray-induced population, five other early flowering mutant lines (10/27, 10/39, 10/60, 10/118 and 10/7) were recovered within 457 to 678 days to first flowering as compared to the control plants flowered after 959.14 days (Figure 9, Table 5). Our results present a workflow for optimizing in vitro growth parameters and irradiation doses to isolate different morphological variations and desirable mutants, such as the early flowering mutant. Figure 10 depicts a complete workflow of the gamma-irradiation-induced mutagenesis system in Dendrobium ‘Emma White’.

## 3. Discussion

The present study describes gamma-radiation-induced mutagenesis and its effect on various stages of in vitro and ex vitro plant growth in Dendrobium ‘Emma White’. Compared to shoots and plantlets, PLBs have been proposed as the most suitable explant to induce variations in different orchid species due to their higher sensitivity toward gamma rays [22,23]. Thus, PLBs of Dendrobium hybrid Emma White were used as explants in the present study. Gamma rays are suitable for obtaining mutants with minor radiation damage [24]. The results revealed a significant effect of gamma irradiation with respect to the overall growth and diverse morphological variations, including early flowering, in ‘Emma White’. The growth response was inversely proportional to increasing radiation doses. However, it is interesting that a lower radiation dose showed enhancement in proliferation and regeneration of PLBs after the radiation treatment (Figure 1). In addition, the results obtained from the study of differential phenotype of in vitro regenerated plantlets indicated the stimulatory effect of low-dose gamma radiation, which is in accordance with the previous report of enhanced growth responses at lower doses of gamma rays [25,26,27,28,29]. This effect may probably be attributed to the induced physiological and hormonal changes resulting in increased growth and development [30]. The effect of higher doses of gamma radiation indicated minimal proliferation and regeneration responses and reduced plant growth, suggesting radiation-induced cellular damage. The result corresponds to a previous report of growth inhibition at gamma doses > 40 Gy in two cultivars of Cymbidium hybrid [31]. A similar decrease in relative growth rate was reported in the growth of PLBs in Cymbidium at doses > 80 Gy [32]. Such a reduction in growth following gamma exposure could be attributed to significant oxidative damage resulting in altered chloroplast structure [25], membrane injuries [32] and a substantial decrease in nucleic acid and soluble protein levels [33], inhibiting metabolism and plant growth [34]. The higher doses alter stomatal morphology, resulting in inadequate gaseous exchange and, hence, lower plant viability [35]. Thus, irradiation at a lower dose is highly recommended in mutation breeding in orchids due to the adverse effects of higher doses on plant growth and survival [35]. In general, explant mortality after irradiation has been observed in many orchid cultivars with higher doses [22,23,36,37], including few other ornamentals [38,39,40].

Nuclear DNA content is suggested as an index of radiation damage in plants [24]. To understand the effect of radiation at the cellular level, nuclear DNA content was estimated based on fluorescence intensity of isolated nuclei using HTCA, which allows rapid high-throughput content screening in a short period over traditional flow cytometry [41]. The DNA content decreased significantly at doses more than 40 Gy, with more pronounced reduction at 80 Gy, suggesting that PLBs are more sensitive to higher doses of gamma rays. Nuclear DNA estimation can be used to measure radiation damage in mutation breeding experiments [35,42,43]. The decrease in DNA content has been related to signal-transduction-induced cell cycle arrest at the G2M phase of cell division [44]. The present study assessed the gamma-radiation-induced frequency of 7%, 9.5% and 51.6% based on morphological variations in regenerants recovered after radiation at 10, 20 and 40 Gy, respectively (Table 2). Such variations are not common in progenies arising from hybridized or self-pollinated orchids [45]. The previous study reported a mutation frequency of 3% induced by ion-bean (C6+) irradiation in two Paphiopedilum species, with no detectable variations by gamma rays [22]. The variegated chlorophyll leaf color mutants (0.4 Gy) and leaf shape mutants (2 Gy) were identified in two Dendrobium species with ion-beam (C6+), respectively [46]. To reduce the occurrence of undesired severe alterations in mutation breeding, the ideal doses that provide high frequency and a spectrum of desirable mutations can be chosen over radiation doses with the highest mutation frequency [47]. Due to their higher heterozygosity, orchids have a high mutation rate and different mutation types in a short cycle [48]. In the case of ornamental plants, single trait changes are generally observed, including harmful and unpredictable changes [49].

Molecular markers are considered an important tool for assessing plant genetic diversity in breeding programs [50]. Among various molecular markers, RAPD is the most common, inexpensive and reliable method for evaluating genetic variability [51], especially for plants such as orchids, where the availability of specific primers is limited. RAPD has been successfully employed in genetic diversity studies of many orchid cultivars [52,53,54,55]. The present study assessed the polymorphism in genomic DNA of mutant plantlets regenerated from gamma-irradiated PLBs of ‘Emma White’ using RAPD markers. A total of thirty-three scorable bands were generated using six RAPD primers, of which twenty-nine bands were polymorphic, with an average polymorphism of 88.89% (Table 3). The results suggest the effectiveness of gamma radiation in inducing higher polymorphism among mutants compared to the control. A previous study reported polymorphism of 46.5% in chemical mutagenesis of Dendrobium ‘Earsakul’ using ISSR markers [56]. As evident from the RAPD banding pattern, the absence of bands could be attributed to various kinds of DNA damage induced by gamma irradiation treatment resulting in generation of genetic diversity among mutants and between mutants and the control. Except for OPAW13, all the primers used in this study showed PIC values > 0.4, indicating informativeness in evaluating and quantifying polymorphism among the mutant population. In addition, the high cophenetic correlation coefficient of 0.82 was observed using the dissimilarity matrix and clustering method, which revealed that the dendrogram precisely preserved the pairwise distances between the original data points, making the dendrogram generated from the UPGMA-based clustering method viable for genetic diversity studies. Cluster analysis delineated mutants of different doses and the control into three distinct clusters, one comprising the control and mutants under 20 and 40 Gy treatment, while the third cluster included mutants under 10 Gy, indicating their potential genetic distinctness from other treatments and the control (Figure 7).

Morphological variations were detected from the in vitro differentiation stage to the ex-vitro stage, with altered leaf structure, multiple serrations, deep notches and asymmetric leaf arrangement in the mutant population (Figure 4 and Figure 8). In a study using gamma-ray-induced mutants of rice, it has been shown that an increase in leaf vein density could result in enhanced photosynthetic efficiency, which indicates that trait alterations can be useful in higher plant productivity [57]. A pre-flowering period in orchids generally requires several years of vegetative growth depending on genera, species and habitat [11,58,59,60,61]. In the case of Dendrobium, it requires a minimum juvenile period of 2 to 5 years for floral induction [62]. In the present study, we isolated an early flowering mutant (10/46) among the 10 Gy-irradiated mutant population after 294 days of ex vitro transfer of in vitro plantlets. Generally, MADS-box genes are involved in floral organ expression and patterning during development in all angiosperms [63]. Low-dose gamma irradiation could have triggered the upregulation of these genes or some other signaling pathways involved in flower development, promoting early flowering. The isolated early flowering mutants are good candidates to further study flowering at the molecular level. A previous study reported overexpression of MADS-box genes (OMADS4 and OMADS1) in transgenic *Arabidopsis* and *Oncidium* cultivars, enabling early flowering [60]. In contrast, delayed flower bud formation to full bloom at 5 Gy gamma radiation treatment was reported in chrysanthemums [64]. We have also observed changes in the floral morphology in terms of in fused sepals and missing petals in the early flower mutant line (10/46) (Supplementary Figure S1). Similar morphological changes due to gamma irradiation influencing the shape of petals, sepals and the lip were also observed with PLBs of ‘Sonia Kai’ hybrid of Dendrobium [65], which resulted in recovery of four commercial mutants [19]. These changes could be due to a significant decrease in stomata size and cellular damage by radiation [36], which can influence the pattern of both growth and proliferation. However, early flowering in orchids after gamma treatment has not been reported, except for *Phalaenopsis aphrodite* treated at 15 Gy [66]. Our results suggest that mutagenesis can be used to isolate morphological mutants and mutants with desirable attributes for further improvement of valuable orchid hybrids such as Dendrobium ‘Emma White’.

## 4. Materials and Methods

### 4.1. Plant Material for Gamma Irradiation Treatment

PLBs of Dendrobium ‘Emma White’ (D. ‘Singapore White’ x D. ‘Joan Kushima’) were used as explant for mutagenesis, which is a complex hybrid derived from five Dendrobium species [9]. The PLBs generated from the shoot tip of ‘Emma White’ were developed previously at the institute (unpublished work). Before gamma radiation treatment, the established PLBs were maintained on Gamborg basal medium [67] supplemented with 2% sucrose, 0.15% activated charcoal (AC), 0.2 mg/L naphthalene acetic acid (NAA), gelled with 0.7% agar and adjusted at pH 5.8. After two weeks, the PLBs were irradiated with five doses of gamma rays (10, 20, 40, 60 and 80 Gy at a dose rate of 32.54 Gy/min) using ^60^Co gamma irradiator (Gamma Chamber 5000, Bhabha Atomic Research Centre, Trombay, Mumbai, India) as per the standard protocols [68]. All irradiated PLBs were transferred onto the fresh basal medium with the same supplementation. Gamborg basal medium was used as a culture medium throughout the experiment. An un-irradiated set of PLBs were maintained as control. The cultures were incubated at 22 ± 2 °C and 65–70% relative humidity with 16 h photoperiod provided by white fluorescent tube lights (Philips, India, 40 w).

### 4.2. Evaluation of Growth Response of Irradiated PLBs

To assess the immediate effect of gamma ray exposure in ‘Emma White’ the fresh weight of PLBs was measured after a month of irradiation treatment. In addition, growth responses to different irradiation doses were examined based on the survival, proliferation and shoot regeneration rate of PLBs, recorded at three and six months after irradiation. Subsequent development of PLBs after irradiation was investigated by recording the total number of days required for PLBs differentiation in terms of leaf primordium initiation. All growth parameters, including survival, proliferation and regeneration, were calculated based on the percentage of the number of responded PLBs to the total number of PLBs cultured. Accordingly, the optimal dose for mutagenesis was evaluated considering the PLBs growth response after irradiation. After every three months interval, PLBs were transferred into the fresh culture medium, allowing continuous growth and development. The experiment was performed with ten biological replicates and 10–12 PLBs per replicate.

### 4.3. High-Throughput Cell Analysis and Estimation of Nuclear DNA Content of PLBs

After five months of gamma irradiation, nuclei were isolated from irradiated and control PLBs for high-throughput cell analysis and estimation of nuclear DNA following the procedure using Tris-MgCl_2_ buffer [69]. The isolated nuclei pellet was re-suspended in 600 µL of propidium iodide staining buffer overnight at 4 °C in dark conditions. The following day, 200 µL of stained nuclei solution was pipetted into a flat bottom corning 96-well plate and centrifuged at 3000 rpm for 6 min. The plate with loaded samples was placed inside the high-throughput cell analyzer (HTCA) (TTP Labtech’s acumen^®^ Cellista, Melbourn, UK), and flow data were analyzed using acumen Cellista software (version 4.2.5.0.69208). The un-irradiated set of PLBs was processed similarly and taken as control. Based on DNA-intercalating fluorescent dye (propidium iodide), FL3 was selected as the standard fluorescence filer (488 nm excitation and 620 ± 30 nm emission) for the present study. The average fluorescence intensity of nuclei generated by this laser scanning imaging cytometer was used to estimate nuclear DNA content. Based on the selected fluorescence gate, nuclei population defined as G1 (gate 1201–1600) and S + G2M (gate 1601–2800) were used to estimate the average percentage of nuclei in the S + G2M phase of the cell cycle. The experiment was performed with six biological replicates (~25 mg of PLBs per replicate), where each replicate was further divided into two technical replicates. Thus, a total of 12 replicates were used for each irradiation treatment.

### 4.4. Random Amplified Polymorphic DNA (RAPD)-Based Divergence Analysis of In Vitro Gamma Mutants

Total genomic DNA was isolated from in vitro young leaves of control and mutant plantlets using a modified cetyl trimethylammonium bromide (CTAB) method [70]. The isolated DNA was checked for its concentration and purity level using Nanodrop spectrophotometer. A preliminary marker level study was conducted using RAPD to analyze radiation-induced genetic diversity in ‘Emma White’ mutants. A total of 28 decamer RAPD primers were selected based on the previous studies [52,71,72,73,74], of which six primers (OPB 12, OPA 4, OPAW 13, OPAW 17, OPB 5, OPF 1) that produced strong and scorable bands were considered for further analysis. The details of the primers are listed in Supplementary Table S2. The 10 µL of polymerase chain reaction (PCR) mix was prepared containing 2x GoTaq^®^ Green Master Mix (5 µL), 10µM primer (1 µL), nuclease free water (2 µL) and DNA sample (50 ng). PCR amplification was performed using Applied Biosystem Veriti 96-well Thermal Cycler with the first cycle of initial denaturation at 94 °C for 5 min, followed by 35 cycles of denaturation at 94 °C for 45 s, annealing temperature (Tm) specific to individual primers (refer Supplementary Table S2) for 1 min, extension at 72 °C for 2 min and a final cycle of extension at 72 °C for 5 min, followed by hold at 4 °C. The DNA amplicons were analyzed using 1.8% agarose gel, and bands were visualized and photographed using gel documentation system UV trans illuminator ECX-F20.M (GeNei).

### 4.5. Morphological Variation Analysis of Putative Mutants at In Vitro and Ex Vitro Stage

The gamma-irradiated regenerated PLBs were continuously monitored for any detectable morphological changes compared to control. Sub-culture was conducted at a regular interval of two months, allowing continuous growth and development of plantlets. Variation frequency was calculated as the percentage of mutants to the total regenerant PLBs and variation spectrum as the total mutant number [31,75]. After 12–13 months of post irradiation in vitro growth, the expression of differential phenotype was recorded in terms of plant height, leaf number, leaf length, leaf width, root number, root length and root width. The well-rooted plantlets were transferred ex vitro for acclimatization and hardening. A separate set of un-irradiated plantlets were maintained as the control for observation. The plantlets were washed with water to remove excess agar attached to roots, rinsed in 1% systemic fungicides (Carbendazin 50% WP, 02 min) and air-dried (10–15 min) and planted in small pots (10 × 7 cm) with coco-peat. The potted plants were kept inside a polyhouse under controlled conditions. After three months, plants were re-potted into a mixture of media containing coco-chips, brick pieces, leaf molds and stone chips (1:2:2:1 ratio). After six months, plants were transplanted into larger pots (15 × 16 cm) with fresh potting mixture. Each hardened plant was numbered accordingly as radiation dose followed by plant number, e.g., 10/1, 10/2, 10/3, 10/4, 10/5, etc. The morphological variations among the putative mutant populations were regularly monitored and flowering traits, such as days to flower bud initiation (DFBI), days to first flowering (DTFF) and days to withering (DTW), were recorded.

### 4.6. Statistical Analysis

The experiments were conducted in a completely randomized design (CRD). The statistical data were analyzed using R version 4.1.2 (Accessed on 1 November 2021). The influence of gamma irradiation on growth response of PLBs was analyzed using one-way ANOVA, followed by Duncan’s Multiple Range Test (DMRT)-based post hoc analysis to determine the significant differences between irradiation treatments. The optimal gamma dose for mutagenesis of Dendrobium cultivar was analyzed using linear regression equation (y = mx + c) in Microsoft Excel, where y is the dependent variable (proliferation and regeneration rate), x is the independent variable (gamma radiation dose), m is the slope and c is the y-intercept, respectively. For RAPD data analysis, amplified bands were scored for the presence (1) or the absence (0) in a binary matrix. NEI72 coefficient-based genetic distance was calculated to generate dissimilarity matrix, and a dendrogram was constructed using unweighted pair group method with arithmetic mean (UPGMA) clustering method in NTSYSpc software, version 2.10 e [76]. The percentage of polymorphism was calculated as the number of polymorphic bands divided by the total number of bands [77]. The polymorphism information content (PIC) value of a marker was calculated using Gene-Calc bioinformatic tools [78].

## 5. Conclusions

The work described here establishes an optimized in vitro mutagenesis method for isolating gamma-radiation-induced mutants in Dendrobium ‘Emma White’ (Figure 10). It will reduce the time required for radiation dose optimization to generate a mutant population with desired traits, especially in orchids. Furthermore, a low radiation dose of 10 Gy showed a significantly profound stimulatory effect on overall growth and early flower development, indicating the usefulness of low doses in mutation breeding. The isolated mutants with economically valuable traits can be used in plant improvement and for further research into functional genes and related signaling pathways that influence early flowering in mutants.

## Figures and Tables

**Table 1:** Fresh weight of protocorm-like bodies of Dendrobium ‘Emma White’ one month after irradiation with gamma rays.

Treatment	Fresh Weight (mg)
Control	648.6 ± 27.4 a
10 Gy	448.2 ± 16.6 b
20 Gy	294.4 ± 14.3 c
40 Gy	237.1 ± 4.9 d
60 Gy	232.0 ± 4.7 d
80 Gy	209.1 ± 5.9 d

The data represent mean values ± standard error. Means with different lower-case letters (a, b, c, d) are significantly different at *p* < 0.05 (Duncan’s Multiple Range Test).

**Table 2:** Variation frequency and variation spectrum of leaf mutants derived from gamma-irradiated protocorms-like bodies (PLBs) of Dendrobium ‘Emma White’.

Gamma Dose	Leaf Margin	Leaf Vein	Leaf Texture	Leaf Apex	Leaf Shape				Leaf Distribution		Leaf Color	Plantlets Regenerated	Total No. of Mutants	Variation Frequency (%)	Variation Spectrum
	T/S	DM	T/R	DN	Lobed	Linear	Ovate	Distorted	LC	T/R	YLWM				
Control	-	-	-	-	-	-	-	-	-	-	-	300	0	-	-
10 Gy	4	4	-	6	-	2	2	-	1	2	-	300	21	7.0	7
20 Gy	5	2	3	3	-	-	1	5	3	-	-	230	22	9.5	7
40 Gy	8	3	2	5	1	-	1	3	2	1	5	60	31	51.6	10

T/S toothed/serrated; DM double midrib; T/R thick/rough; DN deep notch; LC leaf clump; T/R twisted/rolled; YLWM yellow leaves with a white margin. Variation frequency (%) calculated based on morphological variations observed to the total regenerated plantlets.

**Table 3:** Random amplified polymorphic DNA (RAPD) banding pattern analysis in gamma-ray-induced mutant plantlets at doses 10, 20 and 40 Gy.

SI. No.	Primer	Total No. of Bands	Monomorphic	Polymorphic	Polymorphism (%)	PIC
1	OPB12	4	0	4	100	0.48
2	OPA4	4	0	4	100	0.41
3	OPAW13	6	4	2	33.33	0.15
4	OPAW17	6	0	6	100	0.41
5	OPB5	8	0	8	100	0.5
6	OPF1	5	0	5	100	0.5
7	Total	33	4	29	-	-
8	Mean	5.50	0.67	4.83	88.89	0.41

**Table 4:** Genetic dissimilarity matrix from random amplified polymorphic DNA binary data of gamma-ray-induced mutants of Dendrobium ‘Emma White’.

	C_1	C_2	10 Gy_1	10 Gy_2	10 Gy_3	10 Gy_4	20 Gy_1	20 Gy_2	20 Gy_3	40 Gy_1	40 Gy_2	40 Gy_3
C_1	0											
C_2	0.09	0										
10 Gy_1	0.37	0.33	0									
10 Gy_2	0.37	0.33	0.33	0								
10 Gy_3	0.48	0.36	0.38	0.56	0							
10 Gy_4	0.48	0.36	0.46	0.15	0.44	0						
20 Gy_1	0.13	0.13	0.55	0.55	0.61	0.61	0					
20 Gy_2	0.24	0.27	0.46	0.38	0.61	0.43	0.20	0				
20 Gy_3	0.10	0.07	0.32	0.39	0.43	0.50	0.10	0.26	0			
40 Gy_1	0.26	0.19	0.66	0.57	0.37	0.54	0.28	0.38	0.17	0		
40 Gy_2	0.45	0.33	0.63	0.89	0.16	0.61	0.41	0.49	0.33	0.20	0	
40 Gy_3	0.13	0.17	0.51	0.51	0.56	0.48	0.09	0.15	0.15	0.30	0.37	0

**Table 5:** Performance of gamma-radiation-induced early flowering mutants recovered from irradiated protocorm-like bodies of Dendrobium ‘Emma White’ at dose 10 Gy compared with control plants (C).

10 Gy Mutant and Control Lines	DFBI	DTFF	DTW
10/46	224	294	50
10/27	405	457	107
10/39	463	512	79
10/60	595	645	86
10/118	574	669	53
10/7	613	678	81
C/4	906	954	85
C/5(1)	886	923	126
C/5(2)	886	923	76
C/8	910	969	87
C/13	790	862	101
C/11	870	961	52
C/6	1067	1112	79

C: control un-irradiated plants; values within bracket depict flower spike number of the same flower. DFBI: number of days to first bud initiation. DTFF: number of days to first flowering. DTW: number of days to withering.

## Data Availability

All data generated or analyzed during this study are included in this published article (and its Supplementary Materials). Some additional data on de novo whole genome sequence of the gamma-irradiated mutant Dendrobium hybrid cultivar ‘Emma White’ (10 Gy) were deposited with the NCBI with SRA accession number SRR16008784 and Genbank assembly accession GCA_021234465.1, available in the public domain via BioProject ID PRJNA763052. Additional data are available in the GigaScience GigaDB repository.

## Supplementary Materials

The following supporting information can be downloaded at: https://www.mdpi.com/article/10.3390/plants11223168/s1, Figure S1: Comparison of early flower mutant recovered from irradiated PLBs at 10 Gy dose with the flower of ‘Emma White’ mother plant; Table S1: Mean DNA intensity and nuclei count of gamma-irradiated PLBs after five months of irradiation treatment; Table S2: RAPD primers used for genetic study of gamma-irradiated mutants.
