# Supplementary material for: Gamma Radiation Induced In-Vitro Mutagenesis and Isolation of Mutants for Early Flowering and Phytomorphological Variations in Dendrobium ‘Emma White’
Source: Plants (Basel). 2022 Nov 18;11(22):3168. doi: 10.3390/plants11223168 (PMC9697006; doi:10.3390/plants11223168)
Supplement: Supplementary file 1 [file plants-11-03168-s001.zip › plants-1943277-supplementary.pdf]

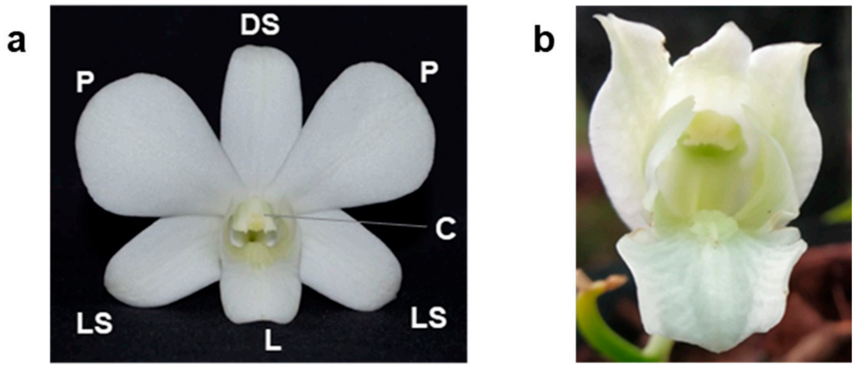

Supplementary Figure S1. Comparison of early flower mutant recovered from irradiated protocorm-like bodies (PLBs) at dose 10 Gy with the flower of 'Emma White' mother plant. Early flowering mutant 10/46 (b) flowered within 294 days of ex vitro growth and was observed with alterations in the floral structure as the dorsal and the lateral sepals fused with no petals. The lip of the mutant flower appeared slightly rounded with wavy edges and longer side lobes compared to the flower of mother plant (a) of Dendrobium 'Emma White' with two petals (P), one dorsal sepal (DS), two lateral sepals (LS), lip (L) and column (C). The mutant plant was numbered according to the radiation dose followed by plant number, i.e., 10 Gy followed by plant number 46 (10/46).

Supplementary Table S1. Mean nuclear DNA intensity and nuclei count of gamma-irradiated protocorm-like bodies (PLBs) after five months of irradiation treatment. The isolated nuclei pellets of irradiated PLBs were resuspended in propidium iodide (PI) staining buffer overnight at 4°C in dark conditions. 200µl of stained nuclei solution was pipetted into a flat bottom corning 96-well plate and centrifuged at 3000 rpm for 6 minutes. PI is a DNA-intercalating fluorescent dye, and its binding with DNA is stoichiometric. Therefore, nuclear DNA content per cell stained with PI is proportional to their FL-3 fluorescence intensity (488 nm excitation and 620±30 nm emission). Mean DNA intensity and percentage of nuclei count at the S+G2M phase of cell cycle were analyzed using high-throughput Cell analyzer (HTCA), and flow data were analyzed using acumen Cellista software. The unirradiated PLBs were taken as control.

Dose	Mean DNA intensity	Nuclei count (%)
Control	1893.58 ± 33.43 a	45.64 ± 2.69 ab
10	1914.50 ± 37.92 a	46.53 ± 3.00 a
20	1824.17 ± 25.04 ab	39.26 ± 1.43 bc
40	1767.50 ± 38.78 bc	38.34 ± 2.80 c
60	1716.58 ± 20.90 c	34.83 ± 2.39 c
80	1601.92 ± 28.38 d	22.09 ± 1.07 d

Supplementary Table S2. Random amplified polymorphic DNA primers used for genetic study of gamma-irradiated mutants of Dendrobium 'Emma White'

Primer code	Sequence (5′- 3′)	G+C Content (%)	Tm (°C)
OPF 01	ACGGATCCTG	60	42
OPF 02	GAGGATCCCT	60	33.7
OPF 03	CCTGATCACC	60	38.8
OPF 04	GGTGATCAGG	60	36
OPF 05	CCGAATTCCC	60	36
OPF 06	GGGAATTCCC	60	40.8
OPD 03	GTCGCCGTCA	70	58
OPB 18	CCACAGCAGT	60	48.4

OPA 1	CAGGCCCTTC	70	48.8
OPD 11	AGCGCCATTG	60	50.3
OPAW 17	TGCTGCTGCC	70	52.7
OPAW 13	CTACGATGCC	60	43.4
OPD 01	ACCGCGAGGG	80	55.8
OPG 15	ACTGGGACTC	60	45.5
OPG 03	GAGCCCTCCA	70	51
OPG 13	CTCTCCGCCA	70	50.3
OPG 14	GGATGAGACC	60	41.8
OPB 05	TGCGCCCTTC	70	52.7
OPB 12	CCTTGACGCA	60	43.4
OPB 17	AGGGAACGAG	60	45.4
OPB 08	GTCCACACGG	70	48.4
OPB 11	GTAGACCCGT	60	45.5
OPB 14	TCCGCTCTGG	70	50.3
OPA 04	AATCGGGCTG	60	43.4
OPZ 04	AGGCTGTGCT	60	50.3
OPU 3	CTATGCCGAC	60	43.4
OPU 10	ACCTCGGCAC	60	51
OPU 13	GGCTGGTTCC	70	48.4
